# Influences of Different Architectures on the Thermodynamic Performance and Network Structure of Aircraft Environmental Control System

**DOI:** 10.3390/e23070855

**Published:** 2021-07-03

**Authors:** Han Yang, Chunxin Yang, Xingjuan Zhang, Xiugan Yuan

**Affiliations:** 1 School of Aeronautic Science and Engineering, Beihang University, Beijing 100191, China; yang_han@buaa.edu.cn (H.Y.); zhangxingjuan@buaa.edu.cn (X.Z.); yuanxg@buaa.edu.cn (X.Y.); 2 Beijing Advanced Discipline Center for Unmanned Aircraft System, Beijing 100191, China

**Keywords:** environmental control system, endoreversible thermodynamic analysis method, refrigeration, pressurization, information entropy, network structure

## Abstract

The environmental control system (ECS) is one of the most important systems in the aircraft used to regulate the pressure, temperature and humidity of the air in the cabin. This study investigates the influences of different architectures on the thermal performance and network structure of ECS. The refrigeration and pressurization performances of ECS with four different architectures are analyzed and compared by the endoreversible thermodynamic analysis method, and their external and internal responses have also been discussed. The results show that the connection modes of the heat exchanger have minor effects on the performance of ECSs, but the influence of the air cycle machine is obvious. This study attempts to abstract the ECS as a network structure based on the graph theory, and use entropy in information theory for quantitative evaluation. The results provide a theoretical basis for the design of ECS and facilitate engineers to make reliable decisions.

## 1. Introduction

Subject to the influence of flight speed and flight altitude, the ambient temperature and pressure change considerably throughout the entire flight envelope [1,2]. The environmental control system (ECS) performs the functions of pressurization, refrigeration and dehumidification simultaneously and provides the guarantee for the health and comfort of the pilot, crew, and passengers. The air cycle system (ACS) is the most commonly used ECS scheme.

Engine bleed air is the power source for ECS in the vast majority of civil and military aircraft. As the aircraft engine is considered as a highly optimized gas generator, there are penalties associated with the extraction of bleed air. Typically, the ratio of the engine power for driving ECS to the aircraft heat load is 10:1 [3]. Vargas and Bejan [4] reported that on a military transport plane such as the C17, the ECS accounts for 64.6% of the engine power at cruise conditions in addition to the irreversible loss of combustion and energy conversion in the engine. In this respect, ECS is one of the largest power consumers among all the nonpropulsive systems [5]. Therefore, it is vital to explore the thermal power conversion mechanism of the ECS to reduce its penalty and improve the aircraft’s energetic response.

An ECS is composed of heat exchangers, the air cycle machine (ACM), and the water separation module. A variety of ECS architectures (such as two-wheel ACS, three-wheel ACS, and four-wheel ACS [3]) have been developed to meet the environmental control requirements of different aircrafts under complex flight conditions. There are obvious differences in the thermodynamic performance among different architectures. Most of the existing research on ECS is focused on the two-wheel ACS. According to the different uses of turbine output power, the two-wheel ACS can be divided into simple and bootstrap types [6]. Vargas and Bejan [4,7] optimized the heat exchanger in the bootstrap ACS. Ordonez and Bejan [8] constructed four simplified ECS-cabin models to evaluate the minimum power requirement for the environmental control of the aircraft. Isabel and Leo [9] optimized the main geometric characteristics of two finned cross-flow heat exchangers involved in a bootstrap ACS. The application of thermo-economics on ECS was also performed [10]. These studies focused on achieving the best performance of a specific ECS. However, different architectures of ECS may change the thermal process and affect the layout of ECS on the aircraft.

There are few experimental studies on the ECS of the aircraft in the open literature, and fewer number of comparative studies on different ECSs because the development and testing of an ECS has a high financial cost. Santos et al. simulated the effects of flight and cabin parameters on the ECS performance [11]. Herber et al. generated many different thermal management system architectures based on graph theory, and evaluated their performance [12]. Conceição et al. developed the thermodynamic models of three-wheel ACS and four-wheel ACS with equation engineering solver [13]. Compared with the existing research, this study significantly improves the analysis method of thermodynamic performance of ECS. The application of endoreversible thermodynamic analysis method (ETM) method to ECS can not only predict the performance of ECS under different working conditions rapidly, but also derive the general solutions of the thermodynamic performance of ECS. The ETM was first applied to a Carnot engine by Curzon and Ahlborn [14]. Compared with the ideal Carnot cycle, the endoreversible cycle considers the temperature difference between the heat source and the working fluid, introduces a time scale and makes the actual thermal process different from the quasistatic process of the ideal cycle. Zhang et al. [15] established a finite-time thermodynamic model for an air Brayton cycle for recovering waste heat from blast furnace slag. Wang et al. [16] compared the performance of air-standard rectangular cycles using finite-time thermodynamics. Amir et al. [17] evaluated and optimized endoreversible combined cycles that considered different heat exchangers. Studies have also been conducted on the Carnot refrigerator [18], supercritical carbon dioxide brayton cycle [19], Stirling engine [20,21,22], diesel engines [23], ocean thermal energy conversion [24], and chemical engine [25]. The relationships among the coefficient of performance (COP), the parameters of components and the environment of a two-wheel ACS [26] and a four-wheel ACS [27] were obtained based on the ETM. The analytical solutions of COP of five types of electrical-driven ECSs were also compared [28]. The solutions derived from ETM are only related to the system architecture, have nothing to do with the operating conditions and the component performance, and have strong universality. However, the existing research mainly focuses on the temperature changes in thermal system especially ECS. As an open cycle, the pressure regulation of ECS is of great significance to ensure human safety in high altitude and low pressure environment.

Additionally, the ECS maintains an open system from disorder to order, and from a low-order degree to a high-order degree, conforming to the description of dissipative structure [29,30]. The theory of information entropy [31] can quantitatively evaluate the order degree of a system [32,33], which have been applied to investigate the organizational structure of the electricity regulatory institution [34], the developmental level and harmonious degree of urban ecosystems [35], etc. The application of information entropy in thermal system will be interesting and worth developing.

In this study, four types of two-wheel ACSs with different architectures are investigated in terms of thermodynamic performance and network structure. The analytical solutions of the COP and pressure of the ECSs are derived based on ETM as algebraic equations, avoiding repeated modeling and calculation. The effects of different bleed conditions, flight conditions and component parameters are discussed. The network structure of the ECSs is constructed according to the graph theory [36,37] and compared based on the structure entropy method (SEM) [38] from the perspective of timeliness and quality of information transmission. This study is conducive to the comparison of the performance of different ECSs, and can reduce the development period and cost considerably. The results provide a solid theoretical basis and a multi-dimensional perspective for ECS evaluation and design, which is conducive to aircraft optimization. With the development of more electric aircraft [39], the ETM and SEM methods have the potential to be applied to the analysis and comparison of engine thermal management system [40]. 

## 2. Methods

### 2.1. ECS Description

**Figure 1:**
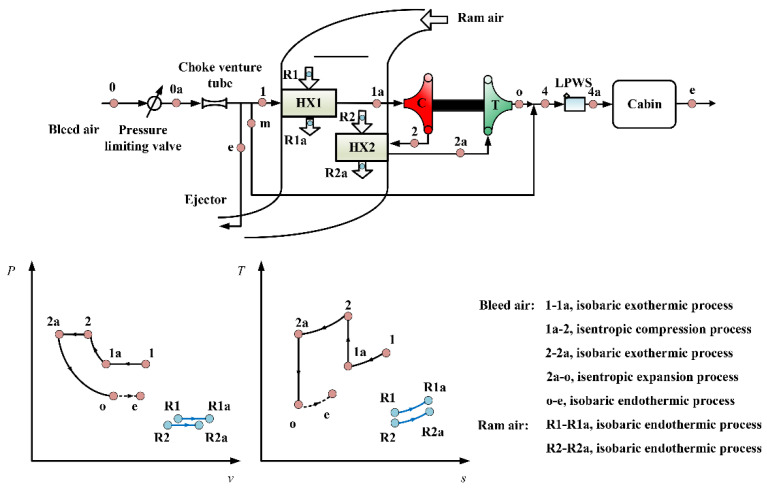
Schematic and thermodynamic process of an ECS.

Figure 1 shows the schematic and thermodynamic process of the ECS of an aircraft that is used as an example to illustrate the analysis process of ETM. The system consists of primary heat exchanger (HX1), secondary heat exchanger (HX2), compressor (C), and turbine (T), etc. Among them, the compressor and turbine are coaxially installed and form the ACM. When the system begins to operate, the high-temperature and high-pressure engine bleed air is regulated to the allowable pressure and proper mass flow through the pressure limiting valve and choke venturi tube, respectively. After passing through the venturi tube, the bleed air is divided three ways: one stream of air is used as the ejection gas to eject the ram air on the cold side of the heat exchangers, the other is used as the bypass air to adjust the cabin temperature by mixing it with low-temperature air at the outlet of the turbine, and the remaining air is used as the air-conditioner working medium to adjust the cabin temperature and pressure. The specific process of air conditioning is as follows: the bleed air enters HX1 to dissipate heat. Subsequently, it enters C for further compression. The high-temperature and high-pressure air dissipates the heat in the HX2. Ram air flows as the heat sinks, and passes through the cold sides of the heat exchangers in parallel. The exhaust air at the hot side of HX2 enters T for expansion and cooling, and then mixes with another part of bleed air. The air then enters the low-pressure water separator (LPWS) to discharge the condensed water. The outlet air of ECS is supplied to the cabin to absorb the heat load and is then discharged from the cabin. In the system, the turbine drives the compressor, and the ram air flows in parallel through the cold sides of HX1 and HX2, named as the parallel type two-wheel bootstrap ECS with low-pressure water separation.

The ECS operates on an open cycle in which air is used as the refrigerant. In this study, the influence of humidity in the air is ignored. In addition, the system is simplified as a parallel type two-wheel bootstrap ACS (PB-ACS) that corresponds to the process between the inlet of HX1 (point 1) and the outlet of T (point 0) in Figure 1. In the heat exchanger, isobaric exothermic and isobaric endothermic processes take place. In the ACM, the air undergoes isentropic compression and expansion.

### 2.2. Endoreversible Thermodynamic Analysis Method (ETM)

#### 2.2.1. Thermodynamic Model 

To obtain the thermal performance of PB-ACS, the thermal process of the system is analyzed based on the ETM. We performed the analysis based on the following assumptions: (a) the air is dry and is modeled as an ideal gas with a constant specific heat capacity $c_{p}$, (b) all the components operate in steady-state conditions. Based on the thermodynamic analysis of each component, the analytical solutions of the temperature and pressure at each point of the ACS are obtained.

- HX1 and HX2

When the aircraft is on the ground or flying at low altitudes with high Mach numbers, the ratio of the mass flow of ram air to that of bleed air in the heat exchangers is generally greater than one. In these circumstances, the definition of the effectiveness of HX1 and HX2 is given by Equations (1) and (2). In addition, the hot and cold sides of the heat exchangers satisfy the heat balance, thus yielding Equations (3) and (4).

(1) $$\eta_{{\mathrm{HX}}_{1}}=\frac{\theta_{1}-\theta_{1a}}{\theta_{1}}$$

(2) $$\eta_{{\mathrm{HX}}_{2}}=\frac{\varphi_{2}-\varphi_{2a}}{\varphi_{2}}$$

(3) $$\theta_{1}-\theta_{1a}=\xi_{1}\theta_{R1a}$$

(4) $$\varphi_{2}-\varphi_{2a}=\xi_{2}\varphi_{R2a}$$

where $\theta_{i}=T_{i}-T_{R1}$, $\varphi_{i}=T_{i}-T_{R2}$, The excess temperature is obtained by considering the temperature of the ram air inlet R1 or R2 as the reference point. $\xi_{1}=\frac{G_{R1}}{G_{b}}$, and $\xi_{2}=\frac{G_{R2}}{G_{b}}$.

- Compressor

The air is compressed in the compressor C. Therefore, the relationship between the outlet and the inlet temperatures of C is obtained by introducing the isentropic efficiency:

(5) $$T_{2}=T_{1a}\left(1+\frac{\pi_{c}^{0.286}-1}{\eta_{c}}\right)$$

We define $\Omega_{c}=\frac{\pi_{c}^{0.286}-1}{\eta_{c}}$, and Equation (5) can be rewritten in the form of Equation (6) listed below.

(6) $$\varphi_{2}=\theta_{1a}\left(\Omega_{c}+1\right)+T_{R1}\left(\Omega_{c}+1\right)-T_{R2}$$

- Turbine

The air is expanded in the turbine. By introducing the isentropic efficiency, the relationship between the outlet and inlet temperature of turbine is as follows:

(7) $$T_{o}=T_{2a}\left[1-\eta_{t}\left(1-\pi_{t}^{-0.286}\right)\right]$$

With the definition of $\Omega_{t1}=\eta_{t1}\left(1-\pi_{t1}^{-0.286}\right)$, the expansion ratio can be expressed as:

(8) $$\pi_{t}={\left(1-\frac{\Omega_{t}}{\eta_{t}}\right)}^{-3.5}$$

where

(9) $$\Omega_{t}=\frac{\varphi_{2a}-\theta_{o}+T_{R2}-T_{R1}}{\varphi_{2a}+T_{R2}}$$

- Constraints

The ACS must meet the constraints of power balance and pressure. The constraint of power balance can be expressed as:

(10) $$\varphi_{2}-\theta_{1a}=\eta_{s}\left(\varphi_{2a}-\theta_{o}\right)$$

where $\eta_{s}$ represents the mechanical efficiency of the shaft on which the impellers of the turbine and compressor are mounted.

The constraint relationship between the outlet pressure and inlet pressure is related to the pressure ratio of C and expansion ratio of T. When the pressure losses in the heat exchangers and pipelines are ignored, the pressure at each point satisfies Equations (11)–(14).

(11) $$\varphi_{2}-\theta_{1a}=\eta_{s}\left(\varphi_{2a}-\theta_{o}\right)$$

(12) $$P_{2}=P_{1a}\pi_{c}$$

(13) $$P_{2a}=P_{2}$$

(14) $$P_{o}=P_{2a}/\pi_{t}$$

- Analytical solutions

The analytical solutions of temperature and pressure at each point of PB-ACS can be derived according to Equations (1)–(14), as shown in Table 1. When the aircraft is flying at high altitudes with low Mach numbers, the ratio of the mass flow of ram air to that of bleed air in the heat exchangers is generally less than one, and the analytical solutions of the state parameters of each point can also be obtained according to the aforementioned process. In other conditions when the ratio of the mass flow is approximately equal to one, either case in Table 1 works.

#### 2.2.2. Validation

The ECS in Figure 1 was tested at different simulated flight conditions [41,42]. In the test, the thermodynamic performance of the ECS under different working conditions such as ambient temperature, flight altitude and flight speed was studied. The test conditions are listed in Table 2 with a total of 40 groups of data, where the hot, standard, and cold represent different ambient temperature, and the specific values can be found in References [1,2]. The temperature and pressure of each state point, the mass flow rates of different airflow branches, and the relative humidity of key points were measured, and the performance of each component can be further obtained. The experimental data under different conditions were shown in detail in the References [41,42].

**Figure 2:**
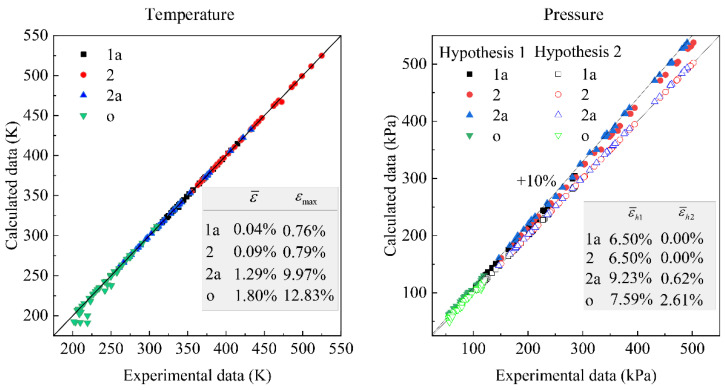
Comparison of experimental and calculated results of PB-ACS.

In addition, in the process of comparison with the test results, according to the equations in Table 1, the theoretical results of the state parameters of each point in the PB-ACS can be obtained by inputting the component characteristics and operating conditions. The comparison between the calculated values and the test data is shown in Figure 2. The definition of the error is shown in Equation (15). The average error $\bar{\varepsilon }=\frac{1}{N}\sum_{i=1}^{N}\varepsilon_{i}$ and maximum error $\varepsilon_{\max}=\max\left\{\varepsilon_{i}\right\}$ can then be obtained.

(15) $$\varepsilon_{i}=\left|\frac{{\mathrm{cal}}_{i}{-\exp}_{i}}{\exp_{i}}\right|$$

As shown in Figure 2, the average error of the temperature at each point between the calculated and the test data is within 1.8% in a wide range, and the average error of the pressure at each point is less than 9.23% without considering the pressure loss in the heat exchanger and pipelines (Hypothesis 1 in Figure 2). The average error can be reduced to smaller than 2.61% after considering the pressure loss in HX1 and HX2 (Hypothesis 2 in Figure 2). The results show that the analytical solutions based on ETM can accurately predict the state of each point.

### 2.3. Structure Entropy Method (SEM)

**Figure 3:**
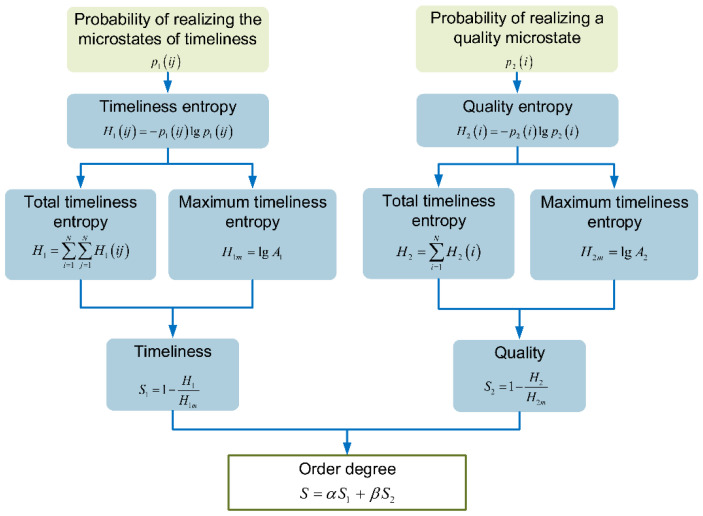
Flowchart of SEM.

As shown in Figure 3, the analysis of SEM includes two aspects: timeliness and quality. The former describes the timeliness of information transmission among nodes in the network structure, and the latter describes the degree of certainty in the information transmission. The order degree of the structure can be obtained by weighting the timeliness and quality. 

As shown in Equation (16), $p_{1}\left(ij\right)$ in Figure 3 is the probability of realizing the microstates of timeliness when the information is transmitted between nodes i and j, and it is related to the path length between the nodes. The path length between a node and its adjacent nodes is defined as 1, which is independent of the actual length of pipe. $p_{2}\left(i\right)$ is the probability of realizing a quality microstate for the *i*-th node, and it is related to the degree of a node, that is, the number of edges connected to each node, as shown in Equation (17).

(16) $$p_{1}\left(ij\right)=L_{ij}/A_{1}$$

(17) $$p_{2}\left(i\right)=k_{i}/A_{2}$$

where i, j represent the number of elements, and i,j=1,2,…,N; $L_{ij}$ is the path length between two nodes; $A_{1}$ denotes the total number of timeliness microstates in the system, and $A_{1}=\sum_{i}\sum_{j}L_{ij}$; $k_{i}$ is the degree of a node; $A_{2}$ denotes the total number of quality microstates, and $A_{2}=\sum_{i}k_{i}$.

## 3. Modeling

### 3.1. Architectures of Different ECSs

**Figure 4:**
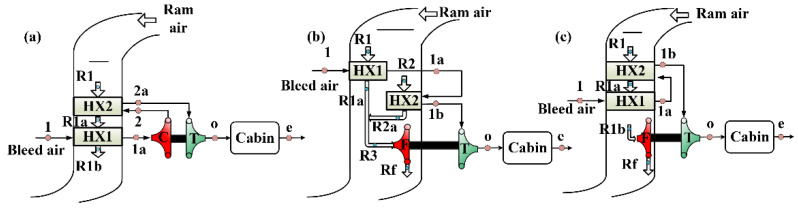
Schematic of ECS with different architectures, (**a**) SB-ACS, (**b**) PS-ACS, and (**c**) SS-ACS.

Considering the scheme of the ACM and the connection mode of the heat exchangers, four types of two-wheel ACSs with different architectures were formed, namely parallel type bootstrap ACS (PB-ACS), serial type bootstrap ACS (SB-ACS), parallel type simple ACS (PS-ACS), and serial type simple ACS (SS-ACS). Except for the PB-ACS in Figure 1, the schematic of the other three ACSs are shown in Figure 4. The turbine-compressor ACM has been adopted in the SB-ACS in Figure 4a as in PB-ACS, and the turbine-fan is used in the PS-ACS and SS-ACS as shown in Figure 4b,c. The heat exchanger in series indicates that a stream of ram air flows through the cold sides of HX2 and HX1 successively. When the ram air is divided into two parts and flows through the cold sides of HX1 and HX2 respectively, this type of heat exchanger is defined as the parallel type heat exchanger. In the early stage, simple ACS was used more often, and the heat exchangers were usually connected in series [43]. Most of the heat exchangers in the bootstrap ACS were connected in parallel.

To simplify the thermal analysis, it is assumed that the thermal state of the ram air in the inlet of the cold side of HX1 and HX2 is consistent, that is, points R1 and R2 in Figure 1 and 4b coincide. Different ACM schemes will change the shape of the thermal cycle, while the main effect of the change of the connection mode of the heat exchanger is embodied in the heat exchange process.

### 3.2. ETM Modeling

The COP is introduced as an indicator of refrigeration performance of ECS, and is defined as:

(18) $$\mathrm{COP}=\frac{Q_{C}}{W_{N}}=\frac{\theta_{e}-\theta_{o}}{\theta_{1}}$$

(19) $$Q_{C}=G_{b}c_{p}(\theta_{e}-\theta_{o})$$

(20) $$W_{N}=G_{b}c_{p}\theta_{1}$$

where *Q_C_* is the cooling capacity of ECS, kW; $c_{p}$ is the specific heat of air, kJ·kg^−1^·K^−1^; $\theta_{e}$ is the excess temperature of cabin exhaust air, K; $W_{N}$ is the power required to drive ECS, kW.

Before analyzing the thermal performance of ECS illustrated in Figure 4, two assumptions are added based on Section 2.2: (1) both the mass flow ratios of HX1 and HX2 are greater than one; (2) the mechanical efficiency of the shaft is equal to one.

The analytical solutions of the outlet temperature, outlet pressure, and COP of the four types of two-wheel ACS with different configurations were derived based on the ETM. The expressions of the outlet temperature and COP of different ACSs are expressed by Equations (21)–(22). The forms of different systems are identical, but each system has unique coefficients. This shows that the COP of ECS is related to the operating conditions and partial component characteristics. It is worth noting that the influences of external and internal factors on the thermal performance of ECS are successfully separated. $T_{R1}$, $\theta_{1}$, and $\theta_{e}$ are the boundary conditions of ECS related to the aircraft and environmental factors, and are classified as the external factors. In addition, the coefficients $Y_{1}$ and $Y_{2}$ are only related to the parameters of the components, such as those of the heat exchanger and compressor or fan. The combination of these component parameters shows the thermal power conversion mechanism of different ECSs. The expressions of the outlet pressure and coefficients $Y_{1}$ and $Y_{2}$ of different ECSs are listed in Table 3.

(21) $$\theta_{o}=Y_{1}T_{R1}+Y_{2}\theta_{1}$$

(22) $$\mathrm{COP}=\left(\theta_{e}-Y_{1}T_{R1}\right)\frac{1}{\theta_{1}}-Y_{2}$$

### 3.3. Network Structure

**Figure 5:**
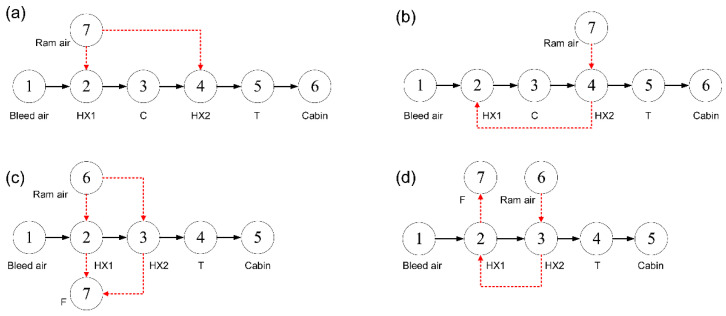
Network structure of different ACSs, (**a**) PB-ACS, (**b**) SB-ACS, (**c**) PS-ACS, (**d**) SS-ACS.

As shown in Figure 5, the network structure of four types of two-wheel ACSs with different architectures is constructed according to the graph theory. The nodes in the graph represent the main elements in the system, and the edges between the nodes represent the connection path between the components, that is, the path of information transmission. The medium of information transmission mainly considers the material flow, i.e., bleed air and ram air, without considering other factors. The network of the two-wheel ACSs presents a chain structure. The bleed air flows through various components in sequence and finally enters the cabin. The existence of fan makes the system have another branch.

## 4. Results and Discussions

### 4.1. Thermodynamic Performance

The refrigeration and pressurization performances of different ECSs were compared in terms of the external and internal responses. The external responses refer to the relationship between the system and the environment, and the internal responses refer to the influence of the parameters of various components on the ECS.

The external factors include the bleed and flight conditions. Considering the actual situation, the range of the values of engine bleed parameter is determined as,

(23) $$400\leq T_{1}\leq 550\;K$$

(24) $$100\leq P_{1}\leq 300\;\mathrm{kPa}$$

The flight conditions refer to the flight altitude and flight speed which determine the stagnation temperature and stagnation pressure of ram air [1,2,44], as shown in Equations (25)–(28). Typically, the flight altitude of civil aircrafts ranges between 0 and 13 km, and the flight speed is between 0 and 0.85 Mach.

(25) $$T_{R1}=T_{h}\left(1+\frac{k-1}{2}Ma^{2}\right)$$

(26) $$T_{h}=T_{h0}-\gamma h$$

(27) $$P_{R1}=P_{h}{\left(1+\frac{k-1}{2}Ma^{2}\right)}^{\frac{k}{k-1}}$$

(28) $$P_{h}=P_{h0}{\left(1-\frac{h}{44.33}\right)}^{\frac{1000g}{\gamma R}}$$

where $T_{h}$ is the atmospheric temperature, K; $T_{h0}$ is the atmospheric temperature at sea level, K; γ is the lapse rate of temperature, and $\gamma =6.5\;K\cdot {\mathrm{km}}^{-1}$; *h* is the flight altitude, km; *Ma* is the flight Mach number; *k* is the adiabatic index, and *k* = 1.4; $P_{h}$ is the atmospheric pressure, kPa; $P_{h0}$ is the atmospheric pressure at sea level, kPa; *g* is gravitational acceleration, and $g\approx 9.81{m/s}^{2}$; *R* is the gas constant, and R≈287J/(kg⋅K).

In the analysis, the values of the parameters of each component are listed in Table 4. The influences of the external and internal factors on the performance of ECS with different architectures were also considered. The simulation tool was Python 3.8.

#### 4.1.1. External Responses

**Figure 6:**
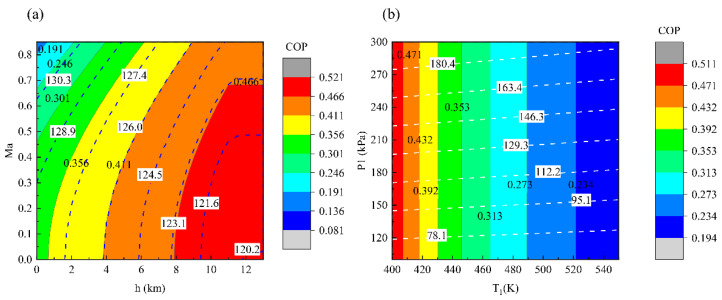
External responses of PB-ACS: (**a**) influence of flight conditions (*T*_1_ = 450 K, *P*_1_ = 200 kPa), and (**b**) influence of bleed conditions (*h* = 0 km, *Ma* = 0).

Figure 6 shows the influences of the external factors on the COP and outlet pressure *P*_o_ of PB-ACS, wherein the solid contours represent the COP, and the dashed contours represent *P*_o_. As shown in Figure 6a, the effects of flight conditions on the COP and *P*_o_ tend to be opposite. In other words, the region with high COP has a low *P*_o_, and vice versa. The influence of the bleed conditions is shown in Figure 6b. The COP of ACS is only affected by the bleed temperature and is independent with the bleed pressure. By contrast, *P*_o_ increases with the increase of bleed pressure and the decrease of bleed temperature, while the effect of the bleed pressure is more obvious.

**Figure 7:**
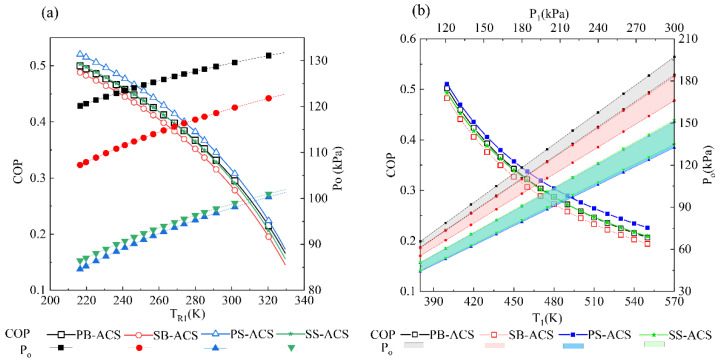
External responses of different ACSs: (**a**) influence of flight conditions (*T*_1_ = 450 K, *P*_1_ = 200 kPa), and (**b**) influence of bleed conditions (*h* = 0 km, *Ma* = 0).

The comparisons for external responses of ACSs with different architectures are illustrated in Figure 7. As shown in Figure 7a, the variation trends of different systems with flight conditions are consistent. With the increase of the stagnation temperature of the ram air, the COP decreases and *P*_o_ increases. Among them, the refrigeration performance of SB-ACS is the worst because the bleed air temperature rises after the secondary pressurization. In addition, a stream of ram air passes through the HX2 and HX1 successively. This is not conducive to the heat transfer of HX1. Therefore, as there is no secondary pressurization and the parallel heat exchanger is beneficial for heat dissipation, the PS-ACS has the highest COP. The pressurization performances of different ACSs are quite varied. The bootstrap ACS is better than the simple ACS in terms of pressurization. It should be noted that changing the temperature of ram air will also affect the outlet pressure of ACS that is the result of internal thermal power transformation. 

Figure 7b shows the influences of the bleed conditions. The COP is only related to the bleed temperature. With the increase of the bleed temperature, the COP of the ECSs decreases gradually. *P*_o_ is related to both the bleed temperature and pressure. Under the same bleed pressure, *P*_o_ decreases with the increase of the bleed temperature. The upper and lower curves of the banded region in Figure 7b correspond to the bleed temperatures of 400 and 550 K, respectively. When the bleed temperature is constant, there is a linear correlation between the *P*_o_ and *P*_1_.

It can be observed from Figure 7 that the pressurization performance is greatly affected by the architectures of ECS when the flight and bleed conditions change. 

#### 4.1.2. Internal Responses

**Figure 8:**
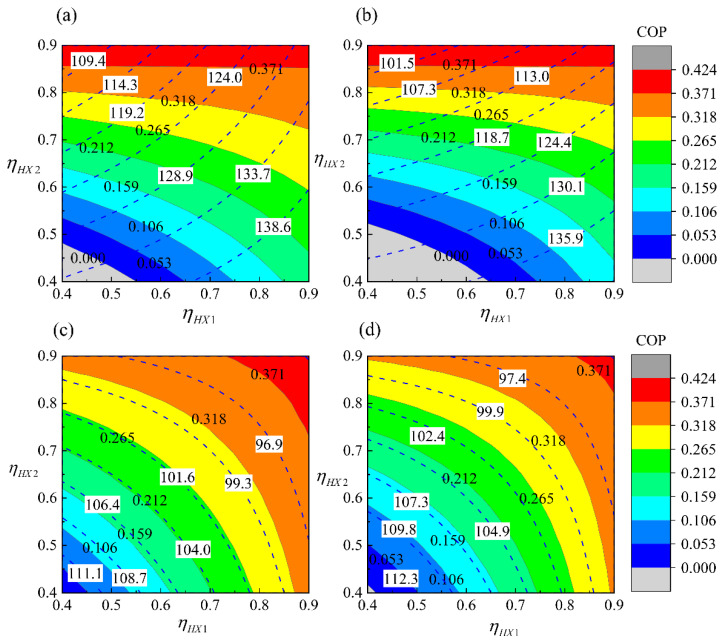
Internal responses of ACSs with the variation of heat exchanger effectiveness: (**a**) PB-ACS, (**b**) SB-ACS, (**c**) PS-ACS, (**d**) SS-ACS (*T*_1_ = 450 K, *P*_1_ = 200 kPa, *h* = 0 km, *Ma* = 0).

The influence of the effectiveness of the heat exchangers is analyzed. Figure 8 shows the connection modes of the heat exchanger have a minor effect on the performances of the ECSs. However, the influence of the air cycle machine is obvious.

As shown in Figure 8a,b, the COP is mainly affected by the effectiveness of HX2, while *P*_o_ is affected by the effectiveness of both HX1 and HX2. Increasing the effectiveness of HX2 will increase the COP but will reduce *P*_o_. Increasing the effectiveness of HX1 is conducive to pressurization. Figure 8c,d shows the effectiveness of HX1 and HX2 are equally important to simple ACSs. The variation trends of refrigeration and pressurization with the effectiveness of heat exchangers are opposite. Increasing the effectiveness of HX1 and HX2 is beneficial to increase the COP, but it reduces the *P*_o_.

In summary, different ACMs coordinate the performance of pressurization and refrigeration. The mechanical energy converted by the turbine in the bootstrap ACS was added in the fresh air through a coaxial compressor to improve the pressurization based on the reduction of the cooling capacity. However, in the simple ACS, the mechanical energy is used to overcome the flow resistance of ram air that could enhance the heat dissipation of the heat exchangers, but the pressurization is not as good as that of the bootstrap ACS.

The effect of the connection mode of heat exchangers is different for the ACSs with different ACMs. The heat exchangers are more suitable for parallel connections in the bootstrap ACS which lead to better refrigeration and pressurization outcomes. For the simple ACS, the parallel connection is beneficial to refrigeration, but has little effect on pressurization. Therefore, for aircraft ECS design, the first task is the selection of the appropriate ACM scheme according to the cooling and pressurization requirements, followed by the arrangement of appropriate layout of the heat exchanger according to the aircraft structure.

### 4.2. SEM

**Figure 9:**
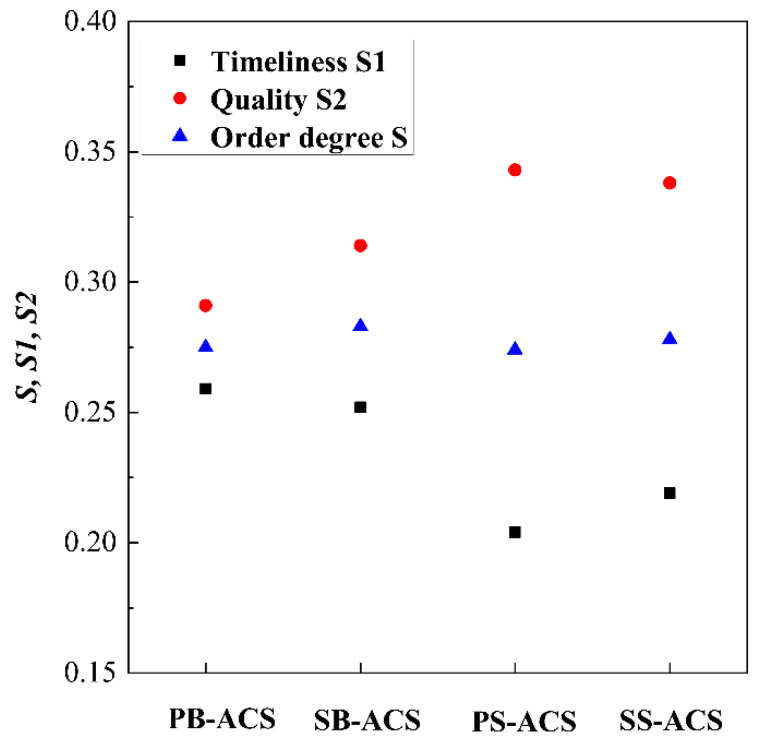
Comparison of network structure of different ECSs.

The results of SEM are also obtained by Python 3.8. As shown in Table 5, the length of different path and degree of each nodes are counted, and other parameters of the structure are obtained. The timeliness, quality, and order degree of different ECS network structures are shown in Figure 9. The weight coefficients α and β are set as 0.5. The ACM scheme mainly affects the dispersion of timeliness and quality, which is closer in the bootstrap ACS. The connection mode of heat exchangers affects the order degree of structure. The serial type ACS has a higher order degree. 

The ETM and SEM quantitatively describe the network structure and function structure of the ACS respectively. Although the order degree cannot be directly compared with the thermal performance parameters, it can evaluate the composition of actual physical system from the perspective of virtual network structure.

## 5. Conclusions

(1) The theoretical results based on ETM are consistent with the test results of ECS in the entire envelope. The average error of the temperature at each point of PB-ACS is within 1.8%. The average error of the pressure at each point is less than 2.61% after considering the pressure loss in HX1 and HX2.

(2) The analytical solutions of the outlet temperature, outlet pressure, and COP of four types of two-wheel ACSs with different architectures are derived based on the ETM. These successfully separate the external and internal factors on the thermal performance of the ECS. The forms of different systems are identical, but each system has unique coefficients.

(3) In the view of thermodynamic performance, the connection mode of the heat exchangers has minor effects on the performance of ECSs, but the influence of the ACM is obvious. In the aspect of network structure, the ACM scheme mainly affects the dispersion of timeliness and quality, and the connection mode of heat exchangers plays a role in the order degree of structure.

This study verifies the feasibility of ETM and SEM. Some useful conclusions have been obtained from the comparative analysis of the ECSs, which can provide suggestions for the design of aircraft engineers. However, the real physical system is a comprehensive network structure of material, energy, and information. We need to further explore the physical connection between the network structure and the actual system, realize the quantitative evaluation of different types of information transfer process, and analyze the relationship between information entropy and thermodynamic entropy.

## Figures and Tables

**Table 1:** Analytical solutions of state parameters at each point of PB-ACS.

$\xi_{1}\geq 1$, $\xi_{2}\geq 1$ (on the ground or fly at low altitudes with high Mach numbers)	
$\theta_{1a}=\left(1-\eta_{{\mathrm{HX}}_{1}}\right)\theta_{1}$	$P_{1a}=P_{1}$
$\varphi_{2}=\left(\Omega_{c}+1\right)\left(1-\eta_{{\mathrm{HX}}_{1}}\right)\theta_{1}+T_{R1}\left(\Omega_{c}+1\right)-T_{R2}$	$P_{2}=P_{1}\pi_{c}$
$\varphi_{2a}=\left(1-\eta_{{\mathrm{HX}}_{2}}\right)\left[\left(\Omega_{c}+1\right)\left(1-\eta_{{\mathrm{HX}}_{1}}\right)\theta_{1}+\left(\Omega_{c}+1\right)T_{R1}-T_{R2}\right]$	$P_{2a}=P_{1}\pi_{c}$
$\theta_{o}=\left(1-\eta_{\mathrm{HX}1}\right)\left[1-\eta_{\mathrm{HX}2}+\left(1-\eta_{\mathrm{HX}2}-\eta_{s}^{-1}\right)\Omega_{c}\right]\theta_{1}-\left(\eta_{s}^{-1}-1+\eta_{\mathrm{HX}2}\right)\left(\Omega_{c}+1\right)T_{R1}+\left(\eta_{s}^{-1}-1+\eta_{\mathrm{HX}2}\right)T_{R2}$	
$P_{o}=P_{1}\pi_{c}{\left(1-\frac{1}{\eta_{t}}\frac{\eta_{s}^{-1}\left(1-\eta_{{\mathrm{HX}}_{1}}\right)\Omega_{c}\theta_{1}+\left[\eta_{s}^{-1}\left(\Omega_{c}+1\right)-1\right]T_{R1}+\left(1-\eta_{s}^{-1}\right)T_{R2}}{\left(\Omega_{c}+1\right)\left(1-\eta_{{\mathrm{HX}}_{2}}\right)\left(1-\eta_{{\mathrm{HX}}_{1}}\right)\theta_{1}+\left(1-\eta_{{\mathrm{HX}}_{2}}\right)\left(\Omega_{c}+1\right)T_{R1}+\eta_{{\mathrm{HX}}_{2}}T_{R2}}\right)}^{3.5}$	
$\xi_{1}<1$, $\xi_{2}<1$ (fly at high altitudes with low Mach numbers)	
$\theta_{1a}=\left(1-\xi_{1}\eta_{{\mathrm{HX}}_{1}}\right)\theta_{1}$	$P_{1a}=P_{1}$
$\varphi_{2}=\left(\Omega_{c}+1\right)\left(1-\xi_{1}\eta_{{\mathrm{HX}}_{1}}\right)\theta_{1}+T_{R1}\left(\Omega_{c}+1\right)-T_{R2}$	$P_{2}=P_{1}\pi_{c}$
$\varphi_{2a}=\left(1-\xi_{2}\eta_{{\mathrm{HX}}_{2}}\right)\left[\left(\Omega_{c}+1\right)\left(1-\xi_{1}\eta_{{\mathrm{HX}}_{1}}\right)\theta_{1}+\left(\Omega_{c}+1\right)T_{R1}-T_{R2}\right]$	$P_{2a}=P_{1}\pi_{c}$
$\theta_{o}=\left(1-\xi_{1}\eta_{\mathrm{HX}1}\right)\left[1-\xi_{2}\eta_{\mathrm{HX}2}+\left(1-\xi_{2}\eta_{\mathrm{HX}2}-\eta_{s}^{-1}\right)\Omega_{c}\right]\theta_{1}-\left(\eta_{s}^{-1}-1+\xi_{2}\eta_{\mathrm{HX}2}\right)\left(\Omega_{c}+1\right)T_{R1}+\left(\eta_{s}^{-1}-1+\xi_{2}\eta_{\mathrm{HX}2}\right)T_{R2}$	
$P_{o}=P_{1}\pi_{c}{\left(1-\frac{1}{\eta_{t}}\frac{\eta_{s}^{-1}\left(1-\xi_{1}\eta_{{\mathrm{HX}}_{1}}\right)\Omega_{c}\theta_{1}+\left[\eta_{s}^{-1}\left(\Omega_{c}+1\right)-1\right]T_{R1}+\left(1-\eta_{s}^{-1}\right)T_{R2}}{\left(\Omega_{c}+1\right)\left(1-\xi_{2}\eta_{{\mathrm{HX}}_{2}}\right)\left(1-\xi_{1}\eta_{{\mathrm{HX}}_{1}}\right)\theta_{1}+\left(1-\xi_{2}\eta_{{\mathrm{HX}}_{2}}\right)\left(\Omega_{c}+1\right)T_{R1}+\xi_{2}\eta_{{\mathrm{HX}}_{2}}T_{R2}}\right)}^{3.5}$	

**Table 2:** List of test conditions.

Flight Altitude(ft)	Atmosphere [1,2]	Mach Number
0 (0 m)	Hot, Standard, Cold	0, 0.145, 0.59, 0.708
6600 (2012 m)	Hot, Standard, Cold	0.168
22,400 (6828 m)	Hot, Standard, Cold	0.244, 0.73
36,100 (11,003 m)	Hot, Standard, Cold	0.352, 0.73, 0.85
42,700 (13,015 m)	Hot, Standard, Cold	0.49, 0.68, 0.85

**Table 3:** Expressions of outlet pressure and coefficients *Y*_1_ and *Y*_2_ for different ECSs.

	Bootstrap ACS
Parallel	$Y_{1}=-\eta_{\mathrm{HX}2}\Omega_{c}$ and $Y_{2}=\left(1-\eta_{\mathrm{HX}1}\right)\left[1-\eta_{\mathrm{HX}2}\left(\Omega_{c}+1\right)\right]$ $P_{o}=P_{1}\pi_{c}{\left(1-\frac{1}{\eta_{t}}\frac{\left(1-\eta_{{\mathrm{HX}}_{1}}\right)\Omega_{c}\theta_{1}+\Omega_{c}T_{R1}}{\left(\Omega_{c}+1\right)\left(1-\eta_{{\mathrm{HX}}_{2}}\right)\left(1-\eta_{{\mathrm{HX}}_{1}}\right)\theta_{1}+\left[\Omega_{c}\left(1-\eta_{{\mathrm{HX}}_{2}}\right)+1\right]T_{R1}}\right)}^{3.5}$
Serial	$Y_{1}=\frac{\eta_{\mathrm{HX}2}\left(\frac{\eta_{\mathrm{HX}1}}{\xi_{1}}-1\right)\Omega_{c}}{1-\left(\Omega_{c}+1\right)\frac{\eta_{\mathrm{HX}1}\eta_{\mathrm{HX}2}}{\xi_{1}}}$ $\;\mathrm{and}\;Y_{2}=\frac{\left(1-\eta_{\mathrm{HX}1}\right)\left[1-\eta_{\mathrm{HX}2}\left(\Omega_{c}+1\right)\right]}{1-\left(\Omega_{c}+1\right)\frac{\eta_{\mathrm{HX}1}\eta_{\mathrm{HX}2}}{\xi_{1}}}$ $P_{o}=P_{1}\pi_{c}{\left(1-\frac{1}{\eta_{t}}\frac{\left(1-\eta_{{\mathrm{HX}}_{1}}\right)\Omega_{c}\theta_{1}+\Omega_{c}\left(1-\frac{\eta_{{\mathrm{HX}}_{1}}\eta_{{\mathrm{HX}}_{2}}}{\xi_{1}}\right)T_{R1}}{\left(\Omega_{c}+1\right)\left(1-\eta_{{\mathrm{HX}}_{2}}\right)\left(1-\eta_{{\mathrm{HX}}_{1}}\right)\theta_{1}+\left[\Omega_{c}\left(1-\eta_{{\mathrm{HX}}_{2}}\right)+1-\left(\Omega_{c}+1\right)\frac{\eta_{{\mathrm{HX}}_{1}}\eta_{{\mathrm{HX}}_{2}}}{\xi_{1}}\right]T_{R1}}\right)}^{3.5}$
	Simple ACS
Parallel	$Y_{1}=-\left(\xi_{1}+\xi_{2}\right)\Omega_{f}$ $\;\mathrm{and}\;Y_{2}=\left(1-\eta_{\mathrm{HX}1}\right)\left(1-\eta_{\mathrm{HX}2}\right)\left(1+\Omega_{f}\right)-\Omega_{f}$ $P_{o}=P_{1}{\left[1-\frac{\Omega_{f}}{\eta_{t}}\left(\frac{\theta_{1}+\left(1+\xi_{1}+\xi_{2}\right)T_{R1}}{\left(1-\eta_{{\mathrm{HX}}_{2}}\right)\left(1-\eta_{{\mathrm{HX}}_{1}}\right)\theta_{1}+T_{R1}}-1\right)\right]}^{3.5}$
Serial	$Y_{1}=-\xi_{1}\Omega_{f}$ $\;\mathrm{and}\;Y_{2}=\frac{\left(1-\eta_{{\mathrm{HX}}_{1}}\right)\left(1-\eta_{{\mathrm{HX}}_{2}}\right)\left(\Omega_{f}+1\right)}{1-\frac{\eta_{{\mathrm{HX}}_{1}}\eta_{{\mathrm{HX}}_{2}}}{\xi_{1}}}-\Omega_{f}$ $P_{o}=P_{1}{\left[1-\frac{\Omega_{f}}{\eta_{t}}\left(\frac{\theta_{1}+\left(1+\xi_{1}\right)T_{R1}}{\frac{\left(1-\eta_{{\mathrm{HX}}_{2}}\right)\left(1-\eta_{{\mathrm{HX}}_{1}}\right)}{1-\frac{\eta_{{\mathrm{HX}}_{1}}\eta_{{\mathrm{HX}}_{2}}}{\xi_{1}}}\theta_{1}+T_{R1}}-1\right)\right]}^{3.5}$

**Table 4:** Parameters of components in the ACS.

	$T_{e},\;K$	$\eta_{HX_{1}}$	$\eta_{HX_{2}}$	$\pi_{c}$	$\eta_{c}$	$\pi_{f}$	$\eta_{f}$	$\eta_{t}$	$\xi_{1}$	$\xi_{2}$
PB-ACS	308	0.8	0.8	1.5	0.75	——	——	0.8	1.1	1.1
SB-ACS	308	0.8	0.8	1.5	0.75	——	——	0.8	2.2	——
PS-ACS	308	0.8	0.8	——	——	1.05	0.25	0.8	1.1	1.1
SS-ACS	308	0.8	0.8	——	——	1.05	0.25	0.8	2.2	——

**Table 5:** Results of SEM.

	$L_{ij}$	$A_{1}$	$H_{1}$	$H_{1m}$	$k_{i}$	$A_{2}$	$H_{2}$	$H_{2m}$
PB-ACS	1,2,3,4,5	37	1.162	1.568	1,2,3	14	0.813	1.146
SB-ACS	1,2,3,4,5	39	1.191	1.591	1,2,3,4	14	0.786	1.146
PS-ACS	1,2,3,4	28	1.151	1.447	1,2,4	16	0.79	1.204
SS-ACS	1,2,3,4	30	1.153	1.477	1,2,4	14	0.759	1.146

## Data Availability

The data presented in this study are openly available in Simulation of Environmental Control System Based on Limited Experimental Data, reference number [42].

## Nomenclature

*A*_1_	total number of timeliness microstates
*A*_2_	total number of quality microstates
*c_p_*	specific heat capacity, kJ·kg^−1^·K^−1^
*g*	gravitational acceleration, m·s^−2^
*G*	mass flow rate, kg·s^−1^
*h*	flight altitude, km
*H*_1_	timeliness entropy
*H*_2_	quality entropy
*H*_1*m*_	maximum timeliness entropy
*H*_2*m*_	maximum quality entropy
*k*	adiabatic index
*k_i_*	node degree
*L_ij_*	path length
*Ma*	mach number
*N*	number of working conditions
*p*	probability
*P*	pressure, kPa
*P_h_*	atmospheric pressure, kPa
*P_h_*_0_	atmospheric pressure at sea level, kPa
*Q_C_*	heat load, kW
*R*	gas constant, J kg^−1^·K^−1^
*s*	entropy, J·K^−1^
*S*	order degree of the system
*S*_1_	timeliness of the system
*S*_2_	quality of the system
*T*	temperature, K
*T_h_*	atmospheric temperature, K
*T_h_*_0_	atmospheric temperature at sea level, K
*v*	volume, m^3^·kg^−1^
*W_N_*	power required to drive ACS, kW
*Y*_1_	coefficient in the analytical solutions of the outlet temperature
*Y*_2_	coefficient in the analytical solutions of the outlet temperature
cal	calculation data
exp	experimental data
**Greek Symbols**	
α	weight coefficients of timeliness
β	weight coefficients of quality
γ	the lapse rate of temperature, K·m^−1^
ξ	ratio between the mass flow rates of the ram air and fresh air
ε	error
$\bar{\varepsilon }$	average error
θ	excess temperature, K
φ	excess temperature, K
$\eta_{HX1}$	effectiveness of primary heat exchanger
$\eta_{HX2}$	effectiveness of secondary heat exchanger
$\eta_{c}$	efficiency of compressor
$\eta_{f}$	efficiency of fan
$\eta_{t}$	efficiency of turbine
$\eta_{s}$	mechanical efficiency of the shaft
$\pi_{c}$	pressure ratio of compressor
$\pi_{f}$	pressure ratio of fan
$\pi_{t}$	expansion ratio of turbine
Ω	customized variable
**Subscripts**	
b	bleed air
c	compressor
e	cabin exhaust air
f	fan
HX	heat exchanger
max	maximum
o	outlet of ACS
R	ram air
t	turbine

## References

[1] Military Standard (1953). MIL-STD-210a. The US Department Non-Standard Atmospheres.

[2] U.S. Government Printing Office (1976). Standard Atmosphere.

[3] Moir I., Seabridge A. (2008). Aircraft Systems Mechanical Electrical and Avionics Subsystems Integration.

[4] Vargas J.V., Bejan A. (2001). Thermodynamic optimization of finned crossflow heat exchangers for aircraft environmental control systems. Int. J. Heat Fluid Flow.

[5] Herzog J. Electrification of the environmental control system. Proceedings of the 25th International Congress of the Aeronautical Sciences.

[6] Linnett K., Crabtree R. What’s next in commercial aircraft environmental control system?. Proceedings of the 23rd International Conference on Environmental Systems, Colorado Springs, Colorado.

[7] Vargas J.V., Bejan A. (2001). Integrative Thermodynamic Optimization of the Environmental Control System of an Aircraft. Int. J. Heat Mass Transf..

[8] Ordonez J.C., Bejan A. (2003). Minimum Power Requirement for Environmental Control of Aircraft. Energy.

[9] Perez-Grande I., Leo T.J. (2002). Optimization of a Commercial Aircraft Environmental Control System. Appl. Therm. Eng..

[10] Leo T.J., Perez-Grande I. (2005). A Thermoeconomic Analysis of a Commercial Aircraft Environmental Control System. Appl. Therm. Eng..

[11] Santos P.P., Andrade C.R., Zaparoli E.L. (2014). A Thermodynamic Study of Air Cycle Machine for Aeronautical Applications. Int. J. Thermo.

[12] Herber D.R., Allison J.T., Buettner R., Abolmoali P., Patnaik S.S. Architecture Generation and Performance Evaluation of Aircraft Thermal Management Systems Through Graph-based Techniques. Proceedings of the AIAA Scitech 2020 Forum.

[13] Conceição S.T., Zaparoli E.L., Turcio W.H.L. Thermodynamic Study of Aircraft Air Conditioning Air Cycle Machine: 3-wheel × 4- wheel. Proceedings of the SAE Brasil 2007 Congress and Exhibit.

[14] Curzon F.L., Ahlborn B. (1975). Efficiency of a Carnot engine at maximum power output. Am. J. Phys..

[15] Zhang Z., Chen L., Yang B., Ge Y., Sun F. (2015). Thermodynamic analysis and optimization of an air Brayton cycle for recovering waste heat of blast furnace slag. Appl. Therm. Eng..

[16] Wang C., Chen L., Ge Y., Sun F. (2016). Comparison of air-standard rectangular cycles with different specific heat models. Appl. Therm. Eng..

[17] Amir G., Said F., Mahdi N.M. (2018). Multi-objective optimization and decision making of endoreversible combined cycles with consideration of different heat exchangers by finite time thermodynamics. Energy Convers. Manag..

[18] Yan Z. (1984). The relation between optimal COP and refrigeration rate of a Carnot Refrigerator. Physics.

[19] Siddiqui M.E., Almitani K.H. (2020). Proposal and Thermodynamic Assessment of S-CO_2_ Brayton Cycle Layout for Improved Heat Recovery. Entropy.

[20] Li Y., He Y., Wang W. (2011). Optimization of solar-powered Stirling heat engine with finite-time thermodynamics. Renew. Energy.

[21] Dai D., Yuan F., Long R., Liu Z., Liu W. (2018). Performance analysis and multi-objective optimization of a Stirling engine based on MOPSOCD. Int. J. Therm. Sci..

[22] Fan S., Li M., Li S., Zhou T., Hu Y., Wu S. (2017). Thermodynamic analysis and optimization of a Stirling cycle for lunar surface nuclear power system. Appl. Therm. Eng..

[23] Gonca G., Sahin B. (2017). Effect of turbo charging and steam injection methods on the performance of a Miller cycle diesel engine (MCDE). Appl. Therm. Eng..

[24] Ikegami Y. (2020). Finite-Time Thermodynamic Model for Evaluating Heat Engines in Ocean Thermal Energy Conversion. Entropy.

[25] Ocampo-García A., Barranco-Jiménez M.A., Angulo-Brown F. (2017). Thermodynamic and thermoeconomic optimization of isothermal endoreversible chemical engine models. Phys. A.

[26] Yang H., Yang C. (2020). Application of Scaling-Endoreversible Thermodynamic Analysis Model to Aircraft Environmental Control System-Methodology Development. Int. J. Refrig..

[27] Yang H., Yang C. (2020). Thermodynamic Characteristics and Order Degree of Air Cycle System. Int. J. Refrig..

[28] Yang H., Yang C. (2020). Derivation and comparison of thermodynamic characteristics of endoreversible aircraft environmental control systems. Appl. Therm. Eng..

[29] Prigogine I. (1978). Time, Structure, and fluctuations. Science.

[30] Yin R. (2016). Theory and Methods of Metallurgical Process Integration.

[31] Shannon C.E. (1948). A mathematical theory of communication. Bell Syst. Tech. J..

[32] Landsberg P.T. (1984). Can entropy and “order” increase together?. Phys. Lett..

[33] Roach T.N., Nulton J., Sibani P., Rohwer F., Salamon P. (2019). Emergent structure in a stochastic model of ecological evolution. Ecol. Model..

[34] Wang Z., He L., Li D. (2019). Assessment of the degree of order in the organizational structure of electricity regulatory institution in China based on shannon entropy. Energy Policy.

[35] Zhang Y., Yang Z., Li W. (2006). Analyses of urban ecosystem based on information entropy. Ecol. Model..

[36] Bondy J.A., Murty U.S.R. (1976). Graph Theory with Applications.

[37] Sedgewick R., Wayne K. (2011). Algorithms.

[38] Yan Z., Qiu W., Chen Z. (1997). Evaluation of system order degree as viewed from entropy. Syst. Eng. Theory Pract..

[39] Kim J., Kwon K., Roy S., Garcia E., Mavris D.N. Megawatt-class Turboelectric Distributed Propulsion, Power, and Thermal Systems for Aircraft. Proceedings of the 2018 AIAA Aerospace Sciences Meeting.

[40] Jafari S., Nikolaidis T. (2018). Thermal Management Systems for Civil Aircraft Engines: Review, Challenges and Exploring the Future. Appl. Sci..

[41] Zhang H., Yuan X., Zhang X. Data processing and fitting of the performance parameters of environmental control system and its application. Proceedings of the 7th National Conference on Environmental Control.

[42] Zhang H. (2005). Simulation of Environmental Control System Based on Limited Experimental Data.

[43] Wang H., Zhang B. (1986). Manual of Foreign Aircraft Environmental Control System.

[44] Shou R., He H. (2004). Aircraft Environmental Control System.

